# Monoaminergic Modulation of Motor Cortex Function

**DOI:** 10.3389/fncir.2017.00072

**Published:** 2017-10-09

**Authors:** Clément Vitrac, Marianne Benoit-Marand

**Affiliations:** ^1^Laboratoire de Neurosciences Expérimentales et Cliniques, INSERM U1084, Poitiers, France; ^2^Laboratoire de Neurosciences Expérimentales et Cliniques, Université de Poitiers, Poitiers, France

**Keywords:** motor cortex, monoamines, dopamine, norepinephrine, serotonin, histamine

## Abstract

Elaboration of appropriate responses to behavioral situations rests on the ability of selecting appropriate motor outcomes in accordance to specific environmental inputs. To this end, the primary motor cortex (M1) is a key structure for the control of voluntary movements and motor skills learning. Subcortical loops regulate the activity of the motor cortex and thus contribute to the selection of appropriate motor plans. Monoamines are key mediators of arousal, attention and motivation. Their firing pattern enables a direct encoding of different states thus promoting or repressing the selection of actions adapted to the behavioral context. Monoaminergic modulation of motor systems has been extensively studied in subcortical circuits. Despite evidence of converging projections of multiple neurotransmitters systems in the motor cortex pointing to a direct modulation of local circuits, their contribution to the execution and learning of motor skills is still poorly understood. Monoaminergic dysregulation leads to impaired plasticity and motor function in several neurological and psychiatric conditions, thus it is critical to better understand how monoamines modulate neural activity in the motor cortex. This review aims to provide an update of our current understanding on the monoaminergic modulation of the motor cortex with an emphasis on motor skill learning and execution under physiological conditions.

## Introduction

**Figure 1:**
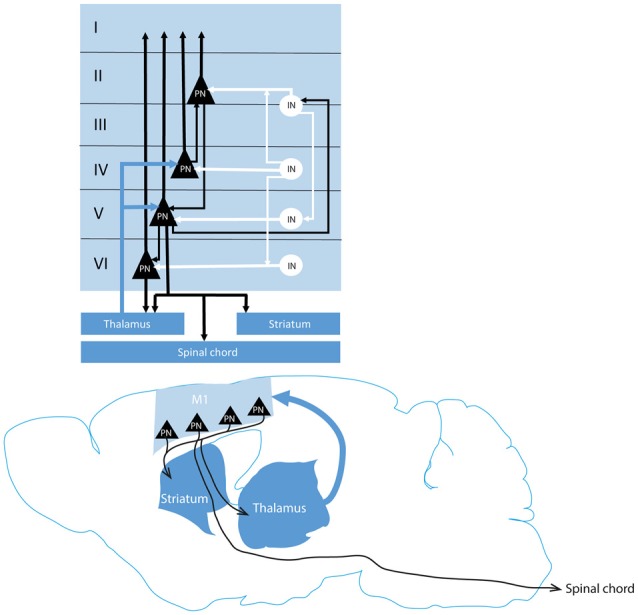
Classical representation of the cortico-subcortical and intracortical motor networks. Thalamic projections to the cortex (blue arrow) reach cortical pyramidal neurons (black triangles) in layer IV and V which contact respectively the layer II/III PN and layer VI PN and inhibitory interneurons (white circle) in the layer II/III. Layer II/III PN contact then PN in the layer V. Layer V and VI PN project to subcortical structures (dark blue). An inhibitory intracortical loop (white) is formed by the inhibitory interneurons in parallel to the excitatory circuitry (black) to prevent epileptiform activity of the cortex. M1, motor cortex; PN, pyramidal neuron; IN, interneuron.

Primary motor cortex (M1) plays a key role in volitional motor control and motor skills learning. M1 neurons are organized in six layers of different populations, about 80% are excitatory glutamatergic neurons and 20% inhibitory γ-amino butyric acidergic (GABA) interneurons (INs; Lev and White, 1997). These neurons are clustered in functional groups and act in a highly specific interconnected local network which processes and transfers the afferent information to cortical and subcortical structures such as the spinal cord and striatum (Zhang and Deschênes, 1997; Lei et al., 2004; Weiler et al., 2008; Anderson et al., 2010; Kiritani et al., 2012; Hira et al., 2013; Oswald et al., 2013; Potjans and Diesmann, 2014; Figure 1). Indeed, M1 receive multiple inputs from other cortical and subcortical structures (Zhang and Deschênes, 1997, 1998; Molyneaux et al., 2007; Kuramoto et al., 2009). Thalamo-cortical projections reach a small band of pyramidal neurons at the layer III/IV border—which shares characteristics with layer IV pyramidal neurons from sensory cortices (Shepherd, 2009; Yamawaki et al., 2014; Barbas and García-Cabezas, 2015). For the sake of simplicity, we will refer to this thin layer of neurons as layer IV. Layer IV neurons project to layer II/III neurons (Potjans and Diesmann, 2014) which contact preferentially the corticostriatal neurons in layer V and to a lesser extent the corticospinal neurons (Cho et al., 2004a,b; Anderson et al., 2010). Corticostriatal neurons send projections to both striatal and to corticospinal neurons (Kiritani et al., 2012). In parallel to this excitatory network, an inhibitory ascendant circuit is recruited to limit the duration of the intracortical activation (Weiler et al., 2008). The somatotopic representation of the body parts is a hallmark of M1 functional organization (Neafsey et al., 1986; Tennant et al., 2011). Experience and training change both the motor maps representations and the excitability of M1 (Nudo et al., 1996; Kleim et al., 1998; Molina-Luna et al., 2008). Synaptic transmission in M1 layer II/III is enhanced in rats after learning a skilled reaching task and synchrony between single neuron activity is increased along training in mice M1 (Rioult-Pedotti et al., 1998, 2000; Peters et al., 2014).

Appropriate behavioral responses to the environment require the ability of networks to integrate environmental clues in order to elaborate appropriate motor responses. The reliability of signal transmission by a neural network is supported by the ability of individual neurons within the network to detect and integrate the signal among the noise. This can be measured by the ratio of signal power to noise power and is referred as signal to noise ratio. Cortical information processing is believed to be sustained by a local excitatory circuit involving monosynaptic connections between PN sustaining persistent activity and an inhibitory network due to non-pyramidal connections onto PN that ensure temporal integration (Durstewitz et al., 2000; Constantinidis and Goldman-Rakic, 2002). This local excitatory/inhibitory network support the increased signal to noise ratio in cortical neurons and is mediated by neuromodulators (Servan-Schreiber et al., 1990; Durstewitz et al., 2000; Kroener et al., 2009).

Neuromodulatory transmitters in the neocortex include monoamines and acetylcholine, this systems share a number of common features and interact locally to shape cortical activity (Gu, 2002). Monoaminergic neurons activity depends on external clues, their role in shaping cortical and subcortical networks activity suggest that they are key players in the fine tuning of adapted motor responses to a given behavioral context.

This review aims to provide an overview of our current knowledge on the role of monoamines on motor cortex function and to integrate all these pieces of information together to propose a hypothesis on cortical monoamine interplay in the regulation of motor response to a given environmental context.

## Dopamine Modulation of Motor Cortex Function

Cortical dopamine (DA) is mainly provided by neurons projecting from the ventral tegmental area (VTA) to the cortex. The mesocortical pathway from the VTA to the prefrontal cortex (PFC) has been well studied, especially in the context of high cognitive processes. The role of DA in executive function and more specifically in motor execution is usually addressed through the study of the basal ganglia modulation by nigrostriatal dopaminergic pathway from the substantia nigra pars compacta to the striatum. However, increasing evidence points to a direct modulation of motor control through a dopaminergic innervation of the motor cortex. DA is synthetized by two enzymatic steps, first the hydroxylation of tyrosine in dihydroxyphenylalanine (DOPA), then the decarboxylation of DOPA by the aromatic amino-acid decarboxylase (AADC) results in DA (Pour revue: Meiser et al., 2013). DA is internalized in vesicles by the vesicular monoamine transporter VMAT2.

Dopaminergic neurons are characterized by their bimodal electrophysiological firing frequencies. As shown in rodents and primates, they exhibit *in vivo* two firing patterns, a tonic firing consisting in an irregular activity at 4 Hz and in response to an appetitive or salient stimulus they fire a burst of 2–6 action potentials (AP) at a frequency of 15 Hz (Grace and Bunney, 1984a,b; Freeman and Bunney, 1987; Kosobud et al., 1994; Mirenowicz and Schultz, 1996; Floresco et al., 2003). Dopaminergic neurons can also code for aversive stimuli by a pause in their tonic firing rate (Schultz, 1998; Eshel et al., 2016). Although some studies suggest that aversion signals modulate DA differentially across different areas, in rodents the three different firing frequencies coding for environmental clues result in different extracellular levels of DA in the striatum due to the kinetics of DA elimination by presynaptic reuptake (Chergui et al., 1994; Benoit-Marand et al., 2000; Floresco et al., 2003; Badrinarayan et al., 2012; Ilango et al., 2012). Computational models suggest that the heterogeneity of extracellular DA release among different regions is due to local differences in autoinhibition feedback (Dreyer et al., 2016). Moreover these models show that the different DA firing patterns result in differential DA receptors occupancy (Dreyer et al., 2010).

### Neuroanatomical Evidence

**Figure 2:**
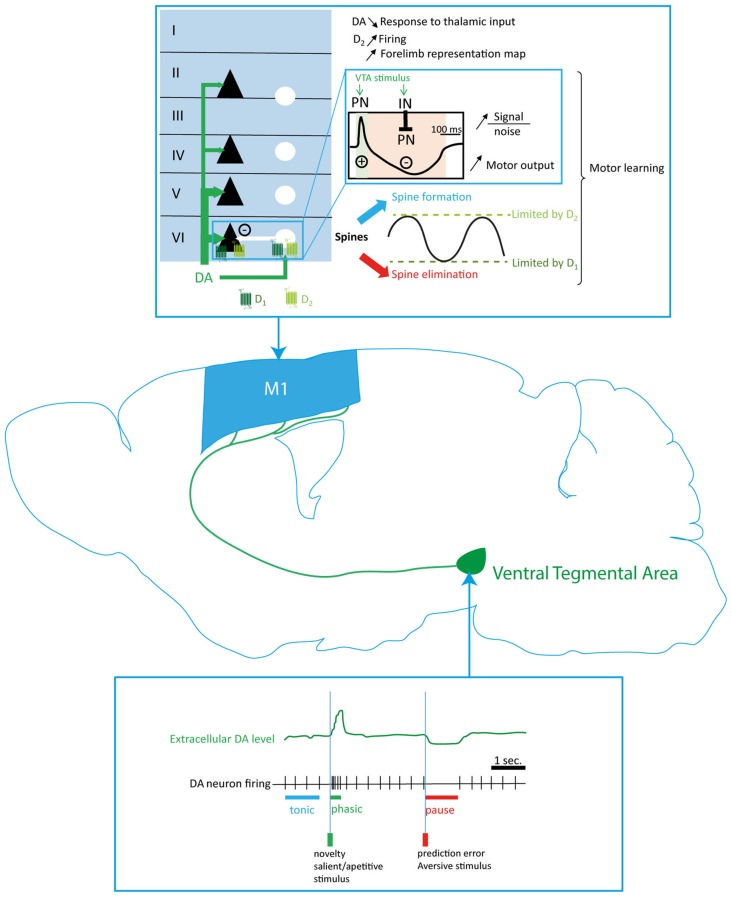
Dopaminergic modulation of the motor cortex function. Dopaminergic innervation of M1 originates in the ventral tegmental area (VTA; green arrows) and preferentially innervates the deep motor cortical layers. Phasic and tonic firing of dopaminergic neurons regulate the dopamine (DA) level in the motor cortex and activate respectively D1 and D2 which are thought to be expressed by the PNs (black triangles) and the INs (white circles) in the motor cortex. Thus, DA innervation exerts a biphasic modulation (blue inlay) of motor cortex neurons to promote motor outputs, increase the signal to noise ratio and regulate the spine turnover necessary to the expression of plasticity and motor learning. DA, dopamine; PN, pyramidal neuron; IN, interneuron.

Early studies have suggested that the motor cortex receives dopaminergic innervation. By using tritiated DA, Descarries et al. (1987) showed the presence of dopaminergic varicosities in layer VI of the entire dorsofrontal cortex in rats, including M1. Later, Gaspar et al. (1991) showed in the human motor cortex the existence of catecholaminergic fibers expressing the tyrosine hydroxylase (TH) but not the dopamine β hydroxylase (DBH), specifically expressed by noradrenergic fibers, thus indirectly demonstrating that human motor cortex is innervated by dopaminergic fibers. Raghanti et al. (2008) showed that TH-expressing fibers innervate M1 in macaques and chimpanzees. Nevertheless, even if TH seems weakly colocalized with DBH in primates’ cortex, these results did not directly demonstrate a dopaminergic innervation of M1 (Gaspar et al., 1989). However, in an elegant study, Hosp et al. (2011) showed that VTA dopaminergic cells project to M1 in rats (Figure 2). The density of the dopaminergic cell bodies innervating the motor cortex in rats decreases in a rostro-caudal gradient inside the VTA (Hosp et al., 2015). These anatomical data suggest that the motor cortex could constitute a territory of limbic and motor interaction. Our group described more specifically the organization of M1 dopaminergic innervation in mice by staining the DA transporter (DAT), specifically expressed by the dopaminergic fibers (Ciliax et al., 1995; Vitrac et al., 2014). We demonstrated that the dopaminergic fibers network in M1 deep layers is dense enough to suggest a modulatory role of M1 activity (Figure 2). Moreover, the dopaminergic fibers preferentially target the forelimb representation map of M1 in rodents supporting the hypothesis that DA innervation from the VTA could modulate M1 neuronal activity (Vitrac et al., 2014; Hosp et al., 2015).

DA tissue levels are detected in the motor cortex. Even though DA concentration in the somatomotor cortex is about 50 times lower than in striatum, the functional implication of DA in cortical regions has been well documented (Godefroy et al., 1991; Awenowicz and Porter, 2002; López-Avila et al., 2004; Schweimer and Hauber, 2006; Hosp et al., 2009; Molina-Luna et al., 2009).

DA acts via five different receptors grouped in two classes, D1 like and D2 like, respectively activating and inhibiting adenylyl cyclase (Jaber et al., 1996). D1-type DA receptors and D2-type DA receptors are expressed in cortical areas of numerous mammalian species, including the motor cortex of rodents (Camps et al., 1990; Mansour et al., 1990; Gaspar et al., 1995). In primates, D1, D2 and D5 mRNAs are found in the motor cortex throughout layers II-V (Huntley et al., 1992). In rodents, D1 receptors are preferentially expressed in layer VI whereas D2 receptors are preferentially expressed in layer V in all the frontal areas, but there is no anatomical evidence indicating whether DA receptors are expressed by PN, interneurons, or both, in M1 (Gaspar et al., 1995). This point is of great importance to understand how DA modulates neuronal activity in this structure.

### Evidence for a Dopaminergic Modulation of Motor Cortex Activity

Direct evidence of the effect of DA on M1 neuronal activity are scarce and conflicting. To date, direct dopaminergic modulation of M1 activity has been essentially studied by *in vivo* electrophysiological techniques. Iontophoretic application of high DA concentration in M1 induced a depressing effect on M1 PN activity in anesthetized rats and a decrease of their response to thalamic and callosal afferences in anesthetized cats (Huda et al., 2001; Awenowicz and Porter, 2002). Furthermore, iontophoretic application of D1 as well as D2 receptor antagonists blocks the depressing effects of DA, demonstrating that DA could inhibit M1 PN activity by a synergistic activation of D1 and D2 receptors (Huda et al., 2001; Awenowicz and Porter, 2002). However, a more recent study showed that a local activation of D2 receptor by a quinpirole injection in the vicinity of recorded PN enhances PN firing rate, demonstrating that DA has an excitatory role via D2 receptor activation (Vitrac et al., 2014). M1 functional outcome can be studied by the extent of its motor representation map as well as by the stimulation threshold required to evoke a motor response. Indeed, peripheral injection of haloperidol, a D2-like receptor antagonist, slows movements and reduces M1 neuronal activity in behaving rats (Parr-Brownlie and Hyland, 2005). Haloperidol administration also results in a decrease of the distal forelimb representation map of M1 in anesthetized rats (Brown et al., 2009). Intracortical injection of a D2, but not D1, receptor antagonist in anesthetized rats confirms the decrease of the distal forelimb representation map of M1 (Hosp et al., 2009). Moreover, Hosp et al. (2009) also demonstrated an increase of both threshold and latency of intracortical-microstimulation (ICMS)-evoked movement after D2 blockade, suggesting that D2 receptors promote neuronal excitability (Figure 2). Interestingly, patch-clamp experiments on PFC slices of adult mice demonstrated that D2 receptor activation with quinpirole selectively prolongs the depolarization of activated PN expressing the D2 receptors by enhancing L-type calcium currents (Gee et al., 2012), confirming that D2 receptor activation can enhance PN activity. However, the behavioral outcome of this modulation is still to be unraveled.

Altogether, these results demonstrate that D2 receptors activation could have an excitatory effect on M1 neuronal activity whereas D1 receptors may not be involved in the modulation of M1 activity. To date, there is no evidence in M1 suggesting a direct effect of D2 receptors on cortico-spinal neurons, a local network effect involving interneurons, or both. However, studies conducted in the PFC show that VTA stimulation increases both PNs and INs firing (Seamans and Yang, 2004; Tseng et al., 2006). As well, the phasic activation of VTA dopaminergic neurons enhances the general excitatory activity as well as gamma oscillations in the secondary motor cortex in mice (Mastwal et al., 2014). Noteworthy, synchronous activation of GABAergic interneurons and PN is responsible for gamma oscillations in cortical structures (Whittington et al., 1995) thus strongly suggesting that DA modulates both neuronal populations in the cortex. If a similar local network effect of VTA stimulation occurs in M1, one could expect as a result a simultaneous activation of PN and interneurons followed by an inhibition of PN due to interneurons feedforward inhibition. An elegant study from Takashima’s group seems to confirm this hypothesis. Indeed, Kunori et al. (2014) demonstrated that VTA stimulation induces a fast excitatory-inhibitory response of M1 neurons resulting in the facilitation of motor output to forelimb muscles. Thus, the dopaminergic modulation from the VTA to M1 could synchronize all neuronal activity in the local network, resulting in an increase of the signal to noise ratio in cortico-spinal neurons (Figure 2).

### Modulation of Motor Cortex Plasticity by DA

DA also modulates M1 plasticity and new motor skill learning. Rioult-Pedotti et al. (1998, 2000) demonstrated that new motor skill learning reinforces M1 horizontal connections within layer II/III. Interestingly, a specific dopaminergic deafferentation of M1 impairs new motor skill learning in rats but not the execution of a previously acquired motor task (Molina-Luna et al., 2009; Hosp et al., 2011). This learning impairment is associated to a loss of the synaptic reinforcement in M1 and is restored by the injection of D1 or D2 receptor agonists suggesting a synergistic involvement of D1-like and D2-like receptors (Molina-Luna et al., 2009; Hosp et al., 2011). Thus, although no role of D1 receptors was described on M1 activity, they are involved in the modulation of M1 plasticity. This was also described *in vitro*, since pharmacological blockade of D1 as well as D2 receptor impairs long term potentiation (LTP) in rat M1 slices, suggesting a synergistic effect of D1 and D2 receptors on plasticity in M1 neural networks (Molina-Luna et al., 2009). This mechanism, challenging the classic view of antagonistic effects of D1 receptor and D2 receptor, is mediated via the non-classical phosphoslipase C (PLC) pathway. Indeed, Rioult-Pedotti et al. (2015) demonstrated that activation of PLC in M1 slices of rat prevents DA receptor blockade-induced impairments of plasticity.

At the structural level, DA differentially regulates spines turnover. In adult mice, *in vivo* D1 receptor blockade with SCH23390 increases spine elimination whereas D2 receptor blockade increases spine formation in M1, suggesting that D1 receptor activation could inhibit spine elimination and that D2 receptor activation could inhibit spine formation (Guo et al., 2015). This is consistent with a previous report in M2 showing that a systemic blockade of D2 receptors by eticlopride enhances the formation of new boutons in M2 induced by a phasic stimulation of the VTA (Mastwal et al., 2014). Nevertheless, these different dopaminergic regulations of spine turnover are not opposed to the synergistic effects on synaptic plasticity described above. Indeed, by setting up a range within which structural plasticity can be expressed, DA could at the same time limit the numbers of irrelevant connections between neurons and prevent aberrant connections (Figure 2). Electrophysiological experiments only showed fewer than 20% of connectivity among PN despite the fact that PN in the output layers of M1 anatomically contact all neighboring neurons (Sjöström et al., 2001; Kalisman et al., 2005; Biane et al., 2015). Interestingly, a sparsely connected neural network is thought to be the basis for a maximal storage capacity. In a neural networks model optimized for memory storage, the fraction of silent synapses or potent synapses is at least 50% (Brunel, 2016). Thus, DA modulation of structural plasticity could contribute to maintain a low fraction of synaptic connections between M1 neurons to optimize memory storage and keep specific functional connections between neurons involved in a relevant behavior. Also, if D1 receptor activity or D2 receptor activity is impaired in M1, the spine turnover could become unstable and structural and synaptic plasticity dampened.

This was confirmed by the demonstration that both D1 and D2 receptors are involved in M1 spine remodeling and synaptic plasticity (Guo et al., 2015). Moreover, local DA depletion in M1 induces spine remodeling in mice as well as motor learning impairment in rats that can be restored by intracortical infusion of levodopa (Molina-Luna et al., 2009; Guo et al., 2015). Altogether, these data suggest that the direct dopaminergic innervation of M1 could regulate structural plasticity.

### Modulation of DA Neuronal Activity by External Stimuli: Implication for Motor Cortex Response to Environmental Clues

Dopaminergic innervation from the VTA to M1 modulates cortical neurons activity and directly impacts M1 synaptic plasticity. This results in an enhancement of neuronal pathways involved in relevant motor outputs to refine new motor skill learning. As such, M1 plays a crucial role in the planning and execution of movements.

DA acts as a key interface between the sensory function and voluntary movements that allow for the selection of motor plans appropriate to the environmental and behavioral contexts. Indeed, dopaminergic neurons are able to switch from a tonic firing pattern to either a pause in firing or a phasic burst of increased activity in response to specific environmental clues (Freeman and Bunney, 1987; Kosobud et al., 1994; Mirenowicz and Schultz, 1996; Schultz, 2010). Thus, VTA dopaminergic neurons mediate signals related to reward and expectation (Eshel et al., 2016; Figure 2). Given that dopaminergic terminals in M1 originates from the VTA, one could assume that a decrease or a pause in dopaminergic tonic firing, such as occurring following the absence of an expected reward, during a fine motor skill learning may decrease excitatory neuronal transmission in the forelimb motor area to prevent the reinforcement of the non-rewarded movement (Hosp et al., 2011; Eshel et al., 2016). Also, according to the classical view of D1 receptor and D2 receptor opposite effects on physiological events, one could assume that a phasic dopaminergic activity arising from the VTA to signal a reward would be crucial to reinforce the successful behavior. Noteworthy, electrical stimulation of the VTA induces a fast M1 neural network depolarization followed by a long duration hyperpolarization, demonstrating that DA can decrease or increase motor cortex activity (Kunori et al., 2014). This biphasic profile is consistent with the changes in the firing rate of the VTA DA neurons in response to a reward omission (Eshel et al., 2016). A cue previously paired with a reward increases instantaneously the firing rate of dopaminergic neurons. When the reward is then omitted, the firing rate of dopaminergic neurons decreases below the baseline rate. Therefore, a behavior could be facilitated or inhibited depending on whether it has been rewarded or not, by a dopaminergic signal in M1.

## Norepinephrine Modulation of Motor Cortex Function

Norepinephrine (NE) is synthetized from DA by the DBH, this enzyme being specifically expressed in the brain by norepinephrinergic fibers. NE is almost exclusively produced and released throughout the brain by a small number of neurons located in a densely packed pontine structure; the Locus Coeruleus (LC; Descarries and Droz, 1970; Swanson and Hartman, 1975). LC neurons exhibit highly ramified dendritic arbors that largely extend beyond the limit of the nucleus (Swanson, 1976; Foote et al., 1983; Shipley et al., 1996). Through this large arborization, NE neurons receive inputs from numerous brain regions including the PFC, the Bed Nucleus of the Stria Terminalis, the Hypothalamus, the dorsal raphe (DR) and the Amygdala (Pickel et al., 1977; Arnsten and Goldman-Rakic, 1984; Peyron et al., 1998; Van Bockstaele et al., 1998, 1999).

The diversity of these inputs makes the LC a relay for information from the autonomic nervous system, neuroendocrine nuclei, stress and limbic circuits, as well as higher order cognitive centers (for review, see Chandler, 2016). Indeed, NE is a key neurotransmitter system for the circadian regulation of arousal. LC neurons tonic activity depends on sleep and wakefulness states, while phasic activity is related to stimuli salience. Through the ability of NE neurons to fire at different rates and among different patterns, this neuromodulatory system participates to a wide range of behaviors such as brain arousal and wakefulness, locomotor activity, anxiety and stress response as well as executive functions and decision making processes (Aston-Jones and Bloom, 1981a,b; Arnsten and Contant, 1992; Rajkowski et al., 1994; Aston-Jones et al., 2001; Carter et al., 2010; McCall et al., 2015; Schiemann et al., 2015).

### Neuroanatomical Evidence

**Figure 3:**
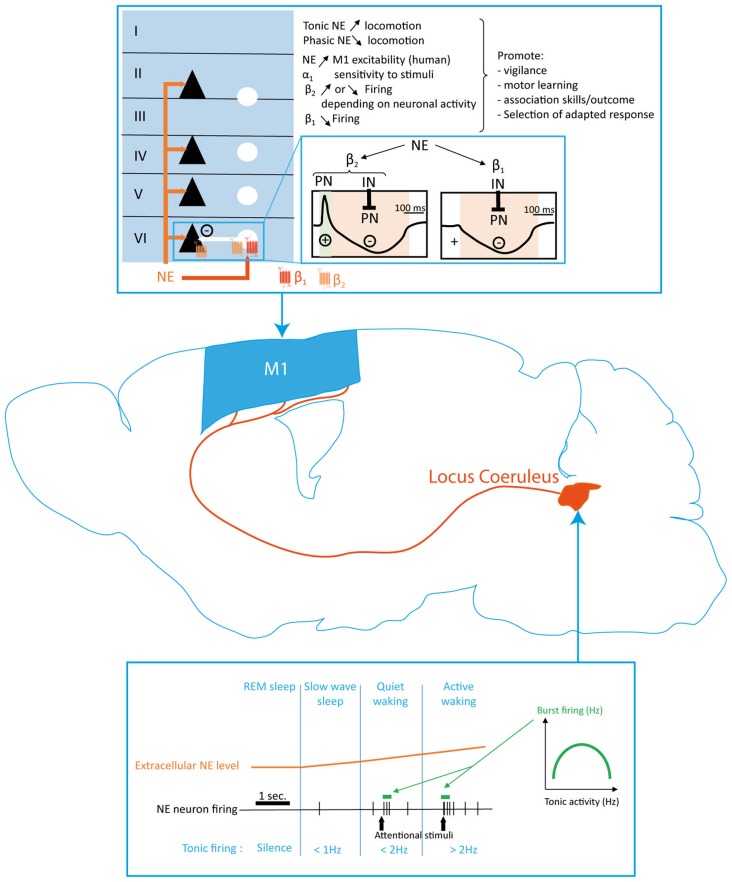
Norepinephrine (NE) modulation of the motor cortex function. NE innervation in M1 originates in the Locus Coeruleus (LC) and terminates homogeneously in all cortical layers. Extracellular NE levels are determined by different neuronal firing frequencies related to the wake/sleep state and firing patterns in response to external stimuli. Tonic and phasic NE release respectively promotes and reduces locomotion. NE exerts its effects in M1 through α_1_, β_1_ and β_2_ receptors located on projection neurons (black triangles) and interneurons (white circles). The differential expression of β_1_ and β_2_ receptors by both neuronal populations (blue inlay) results in two different sequence of excitation/inhibition response in M1 projection neurons. NE modulation of M1 plays a key role in modulating motor response in accordance to the level of vigilance and attention required by the behavioral situation. NE, norepinephrine; PN, pyramidal neuron; IN, interneuron.

The noradrenergic innervation of the brain has been qualitatively described in early studies as a diffuse network of collateralized fibers innervating all neocortical structures, except in the cingulate cortex within which the fibers density has been found higher than in other areas (Fuxe and Ungerstedt, 1968; Jones and Moore, 1977; Jones et al., 1977; Audet et al., 1988). However, it has been shown that NE innervation is segregated in cortical areas (Chandler et al., 2014). LC neurons projecting to the cortex are mostly present in the caudal portion of the nucleus with more than 95% ipsilateral projection pattern (Waterhouse et al., 1983; Figure 3).

Cortical NE is layer specific (Fuxe and Ungerstedt, 1968; Audet et al., 1988), these early studies concluded that NE innervation was more sparse in the superficial than in the deep layers, although this laminar specificity was more evident in primates than in rodents (Morrison and Foote, 1986; Lewis et al., 1987). More recently, the belief that NE fibers are uniformly distributed in rodents throughout the different cortical structures has been challenged. Indeed, Agster et al. (2013) demonstrated using a stereological approach that the density in NE varicosities is higher in the rat PFC than in M1. Moreover, they showed that NE innervation density is higher in layer I than in layer V and VI. This heterogeneous distribution of cortical NE innervation suggests that the amount of NE released during LC activation may vary between regions, thus specific cortical areas could be differentially modulated. Consistent with this idea, an elegant study showed that LC neurons projecting to M1 and PFC are not only anatomically segregated in the LC but also that their physiology differs since M1 projecting neurons exhibit lower firing frequencies and responses to glutamate than those projecting to the PFC (Chandler et al., 2014).

NE effects are mediated by α-adrenoreceptors and β-adrenoreceptors (Summers and McMartin, 1993; Ramos and Arnsten, 2007). Three receptor families have been identified depending on the different intracellular cascades they activate. The α1 adrenoceptors (α1-ARs) activates the PLC and eventually PKC through Gq/11 Protein. The α2 adrenoceptors (α2-ARs) are coupled to a Gi-protein leading to the reduction of cyclic adenosine monophosphate (cAMP). Eventually, three families of β adrenoceptors (β-ARs 1–3) have been characterized, each activating a stimulatory G protein Gs resulting in the elevation of cAMP levels. Noradrenergic receptors α1-ARs, β1-ARs and β3-ARs are postsynaptic whereas α2-ARs and β2-ARs are both pre and post synaptic (O’Donnell, 1993). The three classes of receptors also differ regarding their affinity for NE: α2 (tens of nM) > α1 (300 nM) > β (μM). Noradrenergic receptors are expressed throughout the cortical layers although a clear demonstration of their expression by neuronal subtype in the motor cortex is still lacking. Nearly all the PN in the PFC express β-ARs (Rainbow et al., 1984; Goffinet and De Volder, 1985; Goldman-Rakic et al., 1990; Zhou et al., 2013).

### Evidence of Motor Cortex Functional Modulation by NE

Modulation of the LC neurons activity or of NE levels in the brain modifies cortical activity and excitability in rodents and humans. Microdialysis studies conducted in rat described the release of NE in cortical areas through exocytosis (van Veldhuizen et al., 1990, 1994; Chiti and Teschemacher, 2007). Iontophoretical application of NE in the rat primary somatosensory cortex results in the increase of stimulus-evoked cortical neurons responses (Waterhouse and Woodward, 1980; Waterhouse et al., 1980, 1988). This increase in excitatory synaptic responses following NE application is mediated by α1-ARs (Mouradian et al., 1991; Waterhouse et al., 2000). On the opposite, NE facilitates the inhibitory response to GABA stimulation through β-ARs activation (Waterhouse et al., 1982; Figure 3).

Extracellular recordings performed in M1 layers III to V of anesthetized rats showed that iontophoretic application of β2-ARs agonists either decreased or increased putative pyramidal firing rate whereas β1-ARs agonists decreased pyramidal firing rate (Lukhanina et al., 2003). These cellular effects of NE in M1 suggest a bimodal modulation of firing activity in M1 by NE (Figure 3). Recently, *in vivo* patch clamp recordings demonstrated that topical application of NE antagonists over the forelimb area of M1 induced a hyperpolarization of layer V output neurons and decreased their firing rate when the animal is at rest (Schiemann et al., 2015). This is consistent with human data showing an increase of M1 excitability after blocking NE reuptake (Plewnia et al., 2004). Moreover, NE induces a tonic depolarization of layer V neurons during movement, resulting in the enhancement of M1 neurons firing and promoting contralateral forepaw motor coordination (Schiemann et al., 2015). This study suggests that during movement, NE activate a subpopulation of M1 neurons that are otherwise inhibited.

Altogether, these studies demonstrated that NE inputs to the motor cortex maintains excitability and ameliorates the signal-to-noise ratio in the structure by promoting PN firing during movement in order to ensure a correct movement execution (Figure 3).

### Modulation of Motor Cortex Plasticity by NE

In human, blocking of the NE reuptake enhances motor skill learning indicating that NE can also modulate motor cortex plasticity and suggesting that an increase in NE levels supports LTP (Plewnia et al., 2004). In the visual cortex, Salgado et al. (2012) demonstrated that NE differentially regulates spike timing dependent plasticity (STDP) in the layer II/III. When presynaptic neurons and postsynaptic neurons were stimulated with a negative delay, a high concentration of NE, as a low concentration of NE, triggers LTD. However, for pairings at a short positive delay, a high concentration of NE triggers LTP whereas a low concentration of NE triggers LTD, suggesting that NE is responsible for associative forms of plasticity. Indeed, beta-adrenergic receptors, when activated with agonists, lower the threshold required to induce LTP at synapses between the amygdala and the perirhinal cortex but not at local synapses within the perirhinal cortex (Laing and Bashir, 2014). Amygdala has a key role on emotions processing and motivation, thus implicating NE in modulating plasticity in function of contextual feeling (Janak and Tye, 2015). As such, infusion of β2-ARs agonists in the PFC improved the retention of fear memories in rats (Zhou et al., 2013).

Because α-ARs and β-ARs are coupled to different G proteins like DA receptors, one can imagine that their activation differentially regulates the polarity of plasticity. Indeed, antagonizing α1-ARs led to LTP induction for pairings at negative delay whereas antagonizing β-ARs led to LTD induction at positive delay, suggesting that the formers are responsible for LTD induction whereas the latters are responsible for LTP induction (Salgado et al., 2012). Consistently, bath application of beta-adrenoreceptor agonist in visual cortex slices or PFC slices promotes only LTP at a single cell level with STDP for negative and positive pairings and field potential level with two-input slices preparation whereas bath application of α1-ARs agonist promotes only LTD (Seol et al., 2007; Zhou et al., 2013).

How NE influences motor cortex plasticity has still not been unraveled. In other cortices, NE promotes associative plasticity to link a neuronal pathway to the emotional context during which it has occurred. New motor skill learning is supported by a reinforcement of the synaptic transmission in the layer II/III of M1 (Rioult-Pedotti et al., 1998). Similar to the role of β-ARs on LTP induction in other cortices, their blockade impairs the acquisition of a novel task in rats (Heron et al., 1996). Therefore, mechanisms by which NE modulates plasticity in M1 seem to closely resemble the ones already demonstrated in other cortical areas.

### Modulation of NE Neuronal Activity by External Stimuli: Implication for Motor Cortex Response to Environmental Clues

LC neurons, like other monoaminergic cells, exhibit multimodal firing patterns in response to environmental and behavioral context and NE activity seems to be coordinated with the activity of other neuromodulators (Aston-Jones and Cohen, 2005). NE containing neurons in both anesthetized and unanesthetized rats spontaneously discharge at 0–5 Hz with different tonic frequencies interrupted by bursts of activity (Aston-Jones and Bloom, 1981a,b). In brain slices from young rats, NE neurons exhibit a synchronous oscillatory activity due to an electrotonic coupling between dendrites outside the cell body region (Williams et al., 1984; Ishimatsu and Williams, 1996). This coupling decline with age (Alvarez et al., 2002).

Tonic firing in NE neurons is characterized by low frequency, sustained and highly regular state-dependent firing (Figure 3). Changes in firing discharge rate anticipate changes in behavioral state. During waking, increase in tonic pattern frequency (up to 15 Hz) is elicited by environmental and behavioral stimuli of arousal and attention as demonstrated in cats and rodents (Hobson et al., 1975; Foote et al., 1980; Aston-Jones and Bloom, 1981a,b). Phasic firing pattern is characterized by 2–3 AP followed by a 300–700 ms silent period. This burst of AP occurs during waking in response to salient sensory stimuli with a latency of 15–70 ms. This response presents a habituation related to repeated stimulus presentation (Aston-Jones and Bloom, 1981b; Aston-Jones et al., 1994). Studies conducted in primates and rodents demonstrated that the firing frequency during phasic activity depends on the level of tonic activity in an inverted U-curve dependent relation (Aston-Jones and Bloom, 1981a; Valentino and Foote, 1988; Rajkowski et al., 1994; Figure 3). Unlike DA multimodal activity that is encoded in different DA extracellular levels corresponding to tonic and phasic firing modes, NE extracellular levels linearly follow the frequency of tonic discharge activity (Florin-Lechner et al., 1996; Berridge and Abercrombie, 1999; Figure 3). For instance, salient stimuli, both appetitive and aversive, induce phasic responses in LC neurons as well as an increase in extracellular NE levels in rodents (Foote et al., 1980; Feenstra et al., 2000, 2001; Feenstra, 2000).

These different state-dependent activation patterns of NE neurons result in the modulation of cortical oscillatory activity. For instance, optogenetic inhibition of the LC neurons in mice enhances the slow wave activity of the cerebral cortex characteristic of a sleeping brain, whereas photoactivation of LC neurons decreases the REM sleep cortical activity pattern and awakes mice (Steriade et al., 1993; Carter et al., 2010). This demonstrates that NE controls the general vigilance state of the brain. Interestingly, during cortical slow wave activity, LC neurons fire during the rising phase of the waves, suggesting that NE enhances cortical excitability (Eschenko et al., 2012). Consistent with this hypothesis, inhibition of the NE reuptake by a single oral dose of reboxetine in humans increases M1 excitability (Herwig et al., 2002; Plewnia et al., 2004).

Recent studies suggest that NE neurons are not homogeneous regarding their projection pattern as well as their functional properties. Chandler et al. (2014) demonstrated that PFC and M1 projecting neurons display specific electrophysiological properties depending on their projection target. M1 projecting neurons are less excitable and fire at lower frequencies than PFC projecting neurons, potentially leading to greater NE release in the PFC than in M1.

Multimodal firing patterns of NE neurons allows the generation of responses adapted to the behavioral demand (Schwarz and Luo, 2015). Even though both tonic and phasic firing patterns induce wake from sleep and increase the ratio of wakefulness, only tonic activity increases locomotion and anxiety-like-behavior whereas only phasic NE signaling decreases locomotion (Carter et al., 2010; McCall et al., 2015). Based on their extensive work on LC neuronal firing, Aston-Jones group developed the adaptive gain theory of NE coding (Aston-Jones and Waterhouse, 2016). According to this view, LC neurons would be phasically activated by decision outcome and thus would promote the execution of adaptive behavioral responses whereas high tonic discharge rates would disrupt low priority behaviors and increase flexibility.

## Serotonin Modulation of Motor Cortex Function

Serotonin (5-HT) neurons are found in the Raphe nucleus and innervate almost all the brain structures to promote adaptation of the behavior depending on the environmental context. Thick serotoninergic fibers holding large spherical varicosities are sent by the median raphe whereas thin serotoninergic fibers with small fusiform varicosities originate from the DR (Kosofsky and Molliver, 1987). Neurons of the DR nucleus provide the main source of serotoninergic innervation of the cortex (Jacobs and Azmitia, 1992). They transiently modulate cortical firing rate and firing regularity and pattern depending on environmental context (Jacobs and Azmitia, 1992; Veasey et al., 1997; Ranade and Mainen, 2009; Li et al., 2016). This modulation is mediated by GABAergic, glutamatergic, catecholaminergic and histaminergic inputs from the raphe nucleus itself and structures involved in motor control, cognition and emotions (Jacobs and Azmitia, 1992).

### Neuroanatomical Evidence

**Figure 4:**
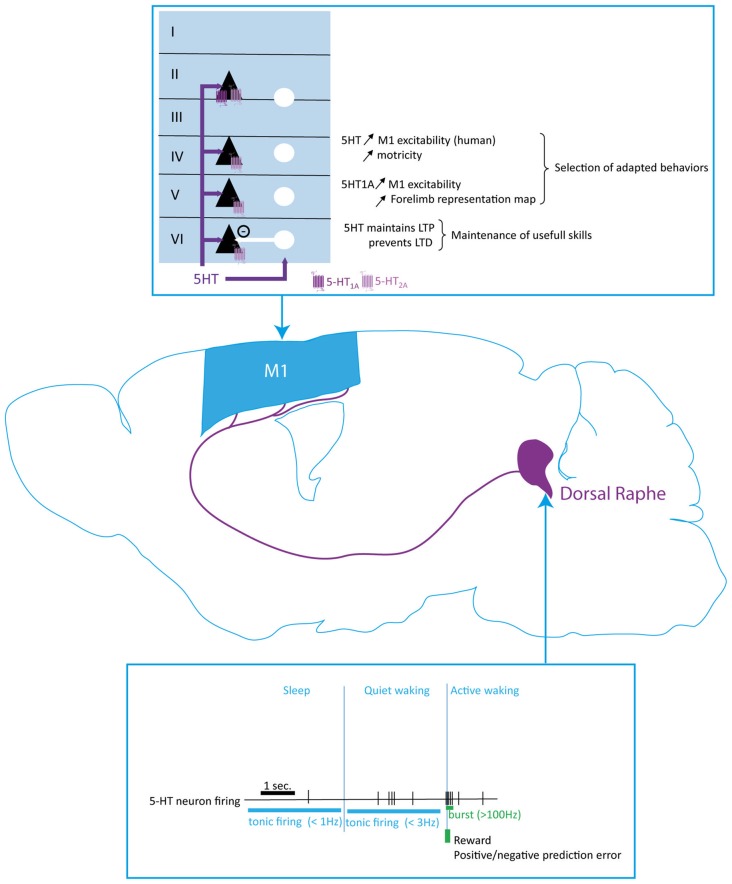
Serotoninergic modulation of the motor cortex function. Serotoninergic neurons from the dorsal raphe (DR) nucleus innervate all cortical layers in M1. The 5-HT neurons different firing frequency and patterns, which are determined by the sleep-wake cycle and response to external stimuli, modifies the extracellular 5-HT level to enhance motor cortex excitability and regulate the maintenance of long term potentiation (LTP). Serotonin plays a key role in the selection of adapted behaviors and maintenance of useful skills. 5-HT, serotonin; PN, pyramidal neuron; IN, interneuron.

A high density of 5-HT fibers has been revealed in early studies in all the frontal cortices and the motor cortex of rats and primates (Vertes, 1991; Wilson and Molliver, 1991). In primates, these fibers predominantly innervate the superficial layers but seem equally distributed throughout the cortical layers in rats (Vertes, 1991; Wilson and Molliver, 1991). Interestingly, the motor cortex is highly innervated by 5-HT fibers in rats whereas the serotoninergic innervation of the motor cortex is lower than in the somatosensory cortex in primates, suggesting a phylogenetical differentiation of the functionality of 5-HT regulation of behaviors (Vertes, 1991; Wilson and Molliver, 1991; Figure 4).

Serotonin acts via 14 receptors, almost all belonging to the superfamily of G protein-coupled receptors. They are classified in seven families referred as 5-HT_1_ to 5-HT_7_ and modulate release of neurotransmitters. The cortical structures express mainly receptors from the 5-HT_1_ and 5-HT_2_ families, with a majority of 5-HT_1A_ (Pazos and Palacios, 1985; Dillon et al., 1991; Celada et al., 2013). 5-HT_1A_ receptor is coupled to a Gi/o protein suggesting an inhibitory role on neuronal activity whereas 5-HT_2A_ is coupled to a Gq protein, suggesting a facilitatory role of neuronal activity.

In the human motor cortex, Joyce et al. (1993) demonstrated that serotoninergic receptors are highly expressed in the superficial layers I/II and weakly expressed in the deepest layers V and VI, matching the fibers distribution showed in primates (Wilson and Molliver, 1991). More precisely, 5-HT_1A_ receptors are mainly expressed in layer II/III whereas 5-HT_2A_ showed a uniform laminar distribution (Joyce et al., 1993; Figure 4). Later, double *in situ* hybridization studies allowed a closer understanding of the organization of the serotoninergic system in the cerebral cortex. At least 50% of the PN and about 30% of the GABAergic neurons express either 5-HT_1A_ and/or 5-HT_2A_ receptors mRNA in the rodent PFC (Santana et al., 2004). Moreover, 80% of the neurons expressing 5-HT_1A_ receptor also express 5-HT_2A_ (Amargós-Bosch et al., 2004). Further studies revealed that each of the receptors are expressed in different compartments of the neurons, suggesting that 5-HT can regulate the synaptic input and action potential generation of the same neuron and strongly influence cortical activity (Jakab and Goldman-Rakic, 1998; Cruz et al., 2004).

### Evidence for Motor Cortex Functional Modulation by 5-HT

Both chronic and acute pharmacological blockade of the 5-HT reuptake by the selective 5-HT reuptake inhibitor (SSRI) paroxetine, in healthy humans increases excitability of the motor cortex as measured by transcranial magnetic stimulation (Loubinoux et al., 2005). Furthermore, during a motor task involving the hand, a single dose of the SSRI fluoxetine enhanced activation of the sensorimotor cortex contralateral to the performing hand (Loubinoux et al., 1999). These data strongly suggest that 5-HT can facilitate motor outputs. Consistent with this, a 5-HT pharmacological depletion in rats decreased the cortical excitability measured by ICMS, caused a motor maps shrinkage and decreased the number of attempts in a skill reaching task (Scullion et al., 2013). Moreover, electrical stimulation of the DR nucleus lowered the movement threshold measured by ICMS, thus increasing cortical excitability through release of 5-HT (Scullion et al., 2013). These effects were blocked by application of a 5-HT_1A_ antagonist and activation of 5-HT_1A_ receptors with 8-OHDPAT increases the size of the forelimb motor area. Further experiments revealed that activation of 5-HT_1A_ increased layer V PN excitation in motor cortex slices and the extent of the motor maps representation (Scullion et al., 2013). These data suggest that 5-HT facilitates motor cortex activation via 5-HT_1A_ receptors (Figure 4). This conclusion is however counterintuitive to the Gi/o-coupling of this receptor and the inhibitory effect reported in other cortical structures. For instance, application of 5-HT by air pressure in PFC mice slices inhibits activity of layer V PN projecting to the pons, confirming a previous *in vivo* study in rats in which stimulation of the DR nucleus inhibits about 2/3 of the PN recorded in the PFC and in the secondary motor areas via 5-HT_1A_ receptors (Puig et al., 2005; Avesar and Gulledge, 2012). Also in the visual cortex, 5-HT enhances GABAergic currents recorded in layer II/III PN (Joo et al., 2015). Expansion of the motor maps is regulated by GABAergic activity in M1 (Chakrabarty and Martin, 2005). Cortical GABAergic neurons express 5-HT_1A_ (Amargós-Bosch et al., 2004; Santana et al., 2004). Therefore, we can hypothesize that the excitatory effects of 5-HT_1A_ in the motor cortex result from inhibition of the GABAergic interneurons. This hypothesis is consistent with *in vivo* data in rodents demonstrating that a stimulation of the DR nucleus inhibits the GABAergic neurons in the PFC via activation of the 5-HT_1A_ receptors (Puig et al., 2010).

Altogether, these results suggest that 5-HT transmission could exert a strong regulation on neuronal networks in the motor cortex. The tonic regular slow pattern of 5-HT neurons could maintain an optimal degree of activity to precisely shape motor cortical maps and promote dexterity and motor output (Figure 4). Accordingly, activation of 5-HT_1A_ receptors regulate the excitatory-inhibitory balance in PFC slices of mice towards excitation (Meunier et al., 2013).

### Modulation of Motor Cortex Plasticity by 5-HT

Consistent with its bidirectional modulation of cortical neurons activity, 5-HT has been reported to enhance or depress synaptic transmission in different cortical structures. However, studies on the role of 5-HT modulation of motor cortex plasticity points toward a facilitatory role of serotoninergic transmission (Figure 4). Indeed, a single dose of the SSRIs paroxetine or citalopram in humans increased motor skill learning and focal plasticity induced by paired associative stimulus (PAS; Loubinoux et al., 2002; Batsikadze et al., 2013). Consistently, chronic citalopram treatment enhances non-focal transcranial direct stimulation (tDCS)-induced plasticity in humans (Nitsche et al., 2009; Kuo et al., 2016). Interestingly, PAS is thought to act through modulation of synapses whereas tDCS modulates the membrane threshold of the neurons, suggesting that 5-HT promotes plasticity by depolarizing neurons and facilitating synaptic transmission in the cortex (Stefan et al., 2000; Nitsche et al., 2008). This is consistent with recent findings showing that activation of 5-HT_1A_ receptors significantly depolarizes layer V PN and lowers the threshold to induce spike in motor cortex slices of rats (Scullion et al., 2013). Furthermore, 5-HT also enhances LTP induction in visual cortex slices of adult rats via enhancement of NMDA currents (Joo et al., 2015).

Interestingly, citalopram also converts tDCS-induced LTD into LTP in human motor cortex and abolishes PAS-induced LTD, indicating that 5-HT prevents motor cortex synapses to express LTD (Batsikadze et al., 2013; Kuo et al., 2016). Furthermore, a prolongation of tDCS-induced plasticity by 5-HT has recently been demonstrated (Kuo et al., 2016). These results strongly suggest that 5-HT transmission in the motor cortex is important for motor memory retention. However, a recent report showed no impairment in motor skills acquisition or in the consequent motor maps enlargement after a 5-HT depletion suggesting that 5-HT has no role or a minor role in motor learning (Scullion et al., 2013).

Altogether, these studies indicate that 5-HT transmission in the motor cortex increases the overall excitability of the neurons to facilitate LTP induction and prevent LTD induction. By these mechanisms, 5-HT could be necessary to retain a newly acquired motor skill. However, 5-HT could have only a minor role on motor skill acquisition *per se*.

### Modulation of 5-HT Neuronal Activity by External Stimuli: Implication for Motor Cortex Response to Environmental Clues

DR nucleus 5-HT neurons strongly innervate the motor cortex and control neuronal activity and plasticity, but seem to have only a minor role on motor learning. Like DA and NE, serotonergic cells display two firing patterns, i.e., tonic and phasic, that code for contextual information (Figure 4). Tonic firing is characterized by a highly regular firing rate at frequencies comprised between 0.1 Hz and 3 Hz in brain slices from rats as well as in behaving cats and rats (Vandermaelen and Aghajanian, 1983; Veasey et al., 1997; Ranade and Mainen, 2009). A fast-firing population of DR neurons has been described, exhibiting tonic firing frequencies up to 17 Hz in rodents (Allers and Sharp, 2003; Kocsis et al., 2006; Hajós et al., 2007). Phasic firing consists in a burst of 2–4 AP with an frequency above 100 Hz (Allers and Sharp, 2003; Kirby et al., 2003; Kocsis et al., 2006; Hajós et al., 2007; Schweimer and Ungless, 2010).

The firing rate shows a strong sleep-wake-arousal cycle with a decreasing firing rate during sleep and increased firing rate during quiet wake as studied in freely moving cats and rodents (Veasey et al., 1997; Ranade and Mainen, 2009). Serotoninergic neurons from the DR nucleus respond to different contextual clues, either by an increase in their firing rate or a shift from tonic to phasic firing pattern. Indeed, during task engagement, the firing rate of these neurons increases and can switch from the tonic to the phasic pattern during motor challenge or reward acquisition (Veasey et al., 1997; Ranade and Mainen, 2009; Li et al., 2016). For instance, 5-HT neurons firing rate increases during odor or sound discrimination to engage in a rewarding task (Ranade and Mainen, 2009; Matias et al., 2017). Interestingly, optogenetic inhibiton of 5-HT neurons in the DR of rats slows reversal learning (Matias et al., 2017). These studies thus suggest that 5-HT codes for cognitive flexibility in the brain. Serotoninergic neurons activity is also enhanced during reward seeking and expectation and a photoactivation of these neurons enhances patience for reward in rats without increasing the saliency of the expected reward (Ranade and Mainen, 2009; Miyazaki et al., 2014; Li et al., 2016). However, no significant changes in 5-HT neurons have been detected after punishment, suggesting that 5-HT encodes reward rather than saliency. In rodents, these neurons also respond to negative or positive violation of the reward prediction error as well as unexpected events (Li et al., 2016; Matias et al., 2017). Altogether, these studies suggest that 5-HT respond to contextual changes to organize selection of a behavior based on the contextual outcome. In the motor cortex, this signal could favor the selection of already acquired motor actions in a repertoire based on their utility. If a motor act is useful and keeps being rewarded as expected or better than expected, 5-HT maintains the heightened synaptic transmission between the neuronal pathways involved. However, when a motor behavior becomes useless because the outcome is worse than expected, 5-HT will promote seeking of a new behavior by disrupting the previously reinforced synaptic pathway and enhancing flexibility in the cortical area.

## Histamine Modulation of Motor Cortex Function

Unlike other monoamines, brain histamine (HA) did not receive attention until the neuroanatomic characterization of histaminergic neurons by the groups of Wada and Panula (Watanabe et al., 1983, 1984; Hayashi et al., 1984; Panula et al., 1984). As a result of the late attention given to HA neurotransmission, only sparse information is available on cortical HA function and even fewer specifically on motor cortex HA modulation. Thus, this section will mostly integrate current knowledge regarding cortical HA.

**Figure 5:**
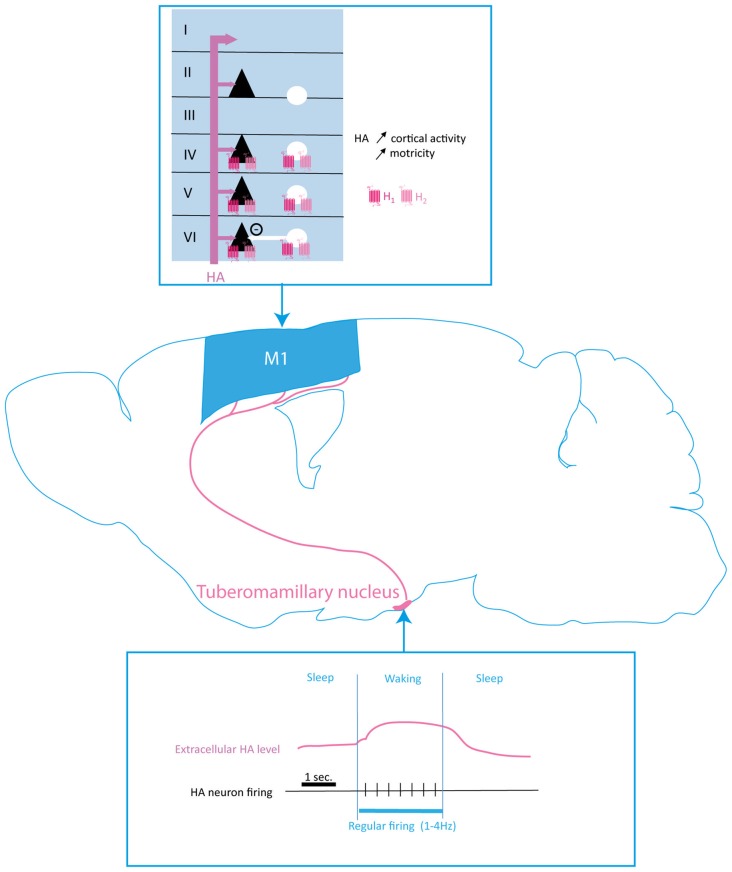
Histaminergic modulation of the motor cortex function. Histamine (HA) input originates in the tuberomamillary nucleus (TMN) and projects to all M1 layers with higher fiber density found in layer I. HA firing and extracellular levels are correlated to sleep/waking rhythm, HA being released tonically during waking. H1 and H2 receptors are both expressed by projection neurons (black triangles) and interneurons (white circles). HA increases cortical activity and motricity. HA, histamine; PN, pyramidal neuron; IN, interneuron.

Histaminergic neurons, located in the tuberomamillary nucleus (TMN), send projections all over the central nervous system in rats, mice and humans (Wilcox and Seybold, 1982; Steinbusch et al., 1986; Wouterlood et al., 1986; Ericson et al., 1987; Panula et al., 1990; Parmentier et al., 2016). Two main bundles of axons send a large arborization to the cortex among other brain structures (Figure 5). Most cortical regions receive a moderately dense or sparse histaminergic input (Haas et al., 2008). The density of HA terminals differs across layers, being denser in layer I whereas evenly distributed across other layers (Panula et al., 1989, 1990; Manning et al., 1996).

HA is synthetized from histidine through the oxidative decarboxylation by histidine-decarboxylase (HDC). The rate of this synthesis depends on the bioavailability of histidine, which is taken up into the neurons through L-amino acid transporters. HA is stored in the soma of neurons and in axons varicosities. Similar to other monoamines, the internalization of HA into vesicles is achieved by the vesicular monoamine transporter VMAT-2 (Merickel and Edwards, 1995; Weihe and Eiden, 2000). HA release is exocytotic and both release and synthesis are under the control of presynaptic H3 autoreceptors located on soma and axonal varicosities (Arrang et al., 1983, 1987; Prast et al., 1994).

The activity of HA neurotransmission follows the circadian rhythm (Figure 5). Indeed, microdialysis and electrophysiology studies showed that HA neurons firing as well as HA release present a marked circadian rhythmicity (Mochizuki et al., 1992). Projections from the preoptic area, the septum, the subiculum, the dorsal tegmentum and the PFC target HA neurons and modulate their activity (Wouterlood et al., 1987, 1988; Wouterlood and Gaykema, 1988). TMN also receives projections from monoaminergic nuclei leading to an increase of HA neurons activity. Indeed, NE projections to TMN, through α2-ARs activation, remove the local GABA inhibition of HA neurons (Stevens et al., 2004).

Cortically-projecting HA neurons recorded in anesthetized rats exhibit a constitutive slow regular firing pattern at a frequency of 1–4 Hz (Reiner and McGeer, 1987). In freely moving animals, the activity of HA neurons follows the circadian rhythm, firing occurs during waking and TMN neurons are silent during sleep (Lin, 2000; John et al., 2004; Takahashi et al., 2006; Parmentier et al., 2016).

HA binds to four metabotropic receptors (H1R to H4R), H4R is mainly found peripherically whereas H1R-H3R are expressed in the brain (Haas et al., 2008; Hu and Chen, 2017). In the cortex, H1R and H2R are found postsynaptically. The H1R is coupled to the Gq/11 protein and phospholipase C and the H2R is coupled to Gs and stimulates adenylyl cyclase resulting in an increase of intracellular cAMP (Haas and Panula, 2003). The H3R is presynaptic on histaminergic neurons as well as on other neurons and postsynaptic in the basal ganglia. H3R is coupled to Gi/o leading to the inhibition of adenylyl cyclase (Hu and Chen, 2017). In the PFC, H3R is also expressed on projecting glutamate neurons (Drutel et al., 2001; Panula and Nuutinen, 2013).

Despite the late anatomical characterization of brain HA, early studies using iontophoretic application of HA agonists suggested the histaminergic modulation of cortical activity (Phillis et al., 1968; Sastry and Phillis, 1976a,b; Haas and Wolf, 1977). Moreover, a recent study investigated the effect of HA on DA, NE and 5-HT transmission in the rat PFC and demonstrated that HA induced increased levels of all three amines aforementioned associated to an increase of neuronal firing restricted to the VTA (Flik et al., 2015).

The circadian activity of HA neurons leads to the modulation of circadian motor activity as well as feeding behaviors (Inzunza et al., 2000; Lozeva et al., 2000; Tuomisto et al., 2001; Haas and Panula, 2003; Meynard et al., 2005; Valdés et al., 2005). Experimental alterations of HA transmission result in motor impairments including a decrease in locomotor activity and a reduction of exploratory behavior in response to a novel environment (Inoue et al., 1996; Toyota et al., 2002; Parmentier et al., 2016). However, this motor impairment could be explained by the well documented innervation of basal ganglia by HA projections.

**Figure 6:**
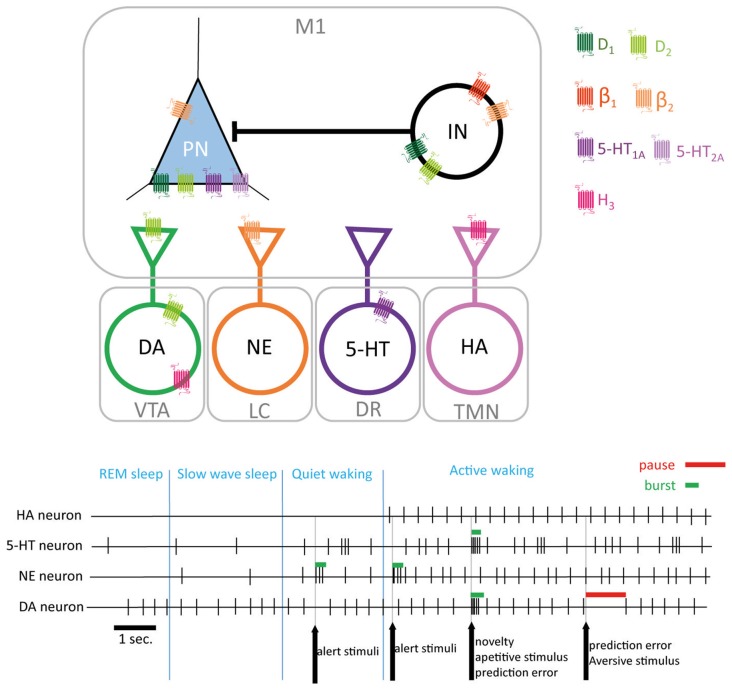
Monoaminergic modulation of M1. Monoamines projecting to M1 interact at pre and post synaptic levels both on pyramidal neurons (PN) and interneurons (IN). Extracellular levels of monoamines are related to sleep/wake states (encoded by tonic firing frequency in HA, 5-HT and NE neurons) and to environmental stimuli supporting attentional/alert information (encoded by bursts or pause in the firing activity by 5-HT, E and DA neurons). DA, dopamine; NE, norepinephrine; 5-HT, serotonin; HA, histamine; M1, primary motor cortex; VTA, ventral tegmental area; LC, locus coeruleus; DR, dorsal raphe; TMN, tuberomamillary nucleus.

Altogether, these data suggest that HA modulates the activity of motor cortex, and could also control other monoamine transmission to cortical neurons (Figures 5, 6). The circadian rhythmicity of HA release as well as its role in wakefulness and sleep state could lead to speculate that HA could play a major role in adjusting cortical activity to match the level of functional outcome required by the attentional state.

## Conclusion

Monoamines are key mediators of arousal, attention and motivation. Their firing pattern enables a direct encoding of different states thus promoting or repressing the selection of actions adapted to the behavioral context. Current knowledge on monoamine in M1 clearly demonstrates their individual role in coding behaviorally relevant sensory information to adjust motor cortex activity and influence the selection of adapted motor responses. In addition, monoamines interact by regulating each other’s activity as well as by sharing common signaling pathways in the target neuron (Figure 6). Motor learning is associated to structural and functional changes in M1, thus the ability of monoamines to modulate M1 plasticity suggest that they can play a major role in the acquisition of new motor skills (Rioult-Pedotti et al., 2000; Kleim et al., 2004; Monfils and Teskey, 2004; Monfils et al., 2005). Other neuromodulatory transmitter systems are known to shape cortical network activity (Gu, 2002). Indeed, the major role of acetylcholine in neocortex modulation has been well reviewed (Lucas-Meunier et al., 2003; Jones, 2004). More recently, the key role of acetylcholine in motor skill learning has been more closely investigated. Indeed, acetylcholine modulates the reorganization of cortical maps following motor training (Conner et al., 2003, 2010; Ramanathan et al., 2009, 2015). Moreover, muscarinic receptors have been shown to be involved in motor cortex activity and plasticity (Desai and Walcott, 2006; Tian et al., 2014; Hedrick and Waters, 2015). Given the interplay that exist between acetylcholine and DA in other brain regions (Calabresi et al., 2006), it would be particularly valuable to investigate the cross-talk between acetylcholine and monoamines in the motor cortex.

Parkinson’s disease (PD) is a progressive neurodegenerative disorder characterized by deficits in motor control and motor learning, although non motor symptoms are also present. The loss of dopaminergic nigrostriatal pathway is the hallmark of PD, this have led to a focus of investigations on the basal ganglia dysfunction in models of nigro-striatal DA depletion. However, the pathological process also involves serotoninergic and histaminergic systems alterations along with an important loss of noradrenergic neurons in the LC (Mann and Yates, 1983; German et al., 1992; Rinne et al., 2002; Marien et al., 2004; Kish et al., 2008; Politis et al., 2010). Moreover, impairments in M1 function have been described in PD patients as well as in PD models (Brown et al., 2009; Guo et al., 2015). In addition to impairments of M1 resulting from a dysfunction of the feedback exerted by basal ganglia on the thalamo-cortical feedback, some local impairments in monoamines have been described. Indeed, a decrease in DA, NE and 5-HT terminals in motor cortical regions in brains from PD patients have been described (Gaspar et al., 1991; Loane et al., 2013). More recently, it was shown that NE as well as 5-HT levels are decreased in M1 from PD patients (Gesi et al., 2000; Marien et al., 2004; Rommelfanger and Weinshenker, 2007). In addition, although no direct evidence indicates any changes in M1 histaminergic system in PD, the decrease of HNMT enzymatic activity observed in some PD patients suggest that a decreased HA degradation might occur leading to alterations of M1 modulation by HA in PD patients (Agúndez et al., 2008; Palada et al., 2012).

Given the increasing hints pointing towards a major role of monoamines in shaping motor responses adapted to behavioral context by controlling the local inhibitory-excitatory network in M1, it appears necessary to better understand the exact role of each monoamines in this highly organized network as well as their reciprocal interaction in physiological as well as pathological conditions. Indeed, the monoamine interplay in regulating M1 function suggests that any pathology unbalancing one of the monoamine could potentially impact homeostasis of all the other monoaminergic systems in the brain, including M1. Thus, the tight regulation of M1 activity and plasticity by monoamines could be altered, leading to impairments in motor learning and context-adapted motor responses.

## Author Contributions

MB-M initiated the writing of the review, designed the organization of the manuscript. Both authors (MB-M and CV) equally contributed to the writing of the manuscript.

## Conflict of Interest Statement

The authors declare that the research was conducted in the absence of any commercial or financial relationships that could be construed as a potential conflict of interest.
